# A novel *SSR4* variant associated with congenital disorder of glycosylation: a case report and related analysis

**DOI:** 10.3389/fgene.2024.1402883

**Published:** 2024-07-17

**Authors:** Wenqiang Sun, Xinyun Jin, Xueping Zhu

**Affiliations:** Department of Neonatology, Children’s Hospital of Soochow University, Suzhou, China

**Keywords:** SSR4 gene, congenital disorders of glycosylation, developmental delay, feeding difficulty, X-linked disease

## Abstract

**Introduction:**

Congenital disorders of glycosylation (CDG) refer to monogenetic diseases characterized by defective glycosylation of proteins or lipids causing multi-organ disorders. Here, we investigate the clinical features and genetic variants of *SSR4*-CDG and conduct a preliminary investigation of its pathogenesis.

**Methods:**

We retrospectively report the clinical data of a male infant with early life respiratory distress, congenital diaphragmatic eventration, cosmetic deformities, and moderate growth retardation. Peripheral blood was collected from the case and parents, genomic DNA was extracted and whole-exome sequencing was performed. The mRNA expression of *SSR4* gene was quantified by Real-time Quantitative PCR. RNA sequencing analysis was subsequently performed on the case and a healthy child.

**Results:**

Whole-exome sequencing of the case and his parents’ genomic DNA identified a hemizygous c.80_96del in *SSR4*, combined with the case’s clinical features, the diagnosis of CDG was finally considered. In this case, the expression of *SSR4* was downregulated. The case were present with 1,078 genes downregulated and 536 genes upregulated. *SSR4* gene expression was significantly downregulated in the case. Meanwhile, gene set enrichment analysis (GSEA) revealed that *SSR4*-CDG may affect hemostasis, coagulation, catabolism, erythrocyte development and homeostatic regulation, and muscle contraction and regulation, etc. Improvement of growth retardation in case after high calorie formula feeding and rehabilitation training.

**Conclusion:**

Our study expanded the *SSR4*-CDG variant spectrum and clinical phenotype and analyzed pathways potentially affected by *SSR4*-CDG, which may provide further insights into the function of *SSR4* and help clinicians better understand this disorder.

## 1 Introduction

Congenital disorders of glycosylation (CDG) refer to monogenetic diseases characterized by defective glycosylation of proteins or lipids causing disorders of multiple organs. The symptoms typically include appearance deformity, abnormal fat distribution, coagulation disorders, neurodevelopmental disorders, and endocrine deficiency (Cylwik et al., 2013a; Cylwik et al., 2013b; Freeze et al., 2014b; Jaeken and Peanne, 2017; Ng and Freeze, 2018). Of the more than 150 kinds of CDGs identified in various glycosylation paths to date, including N-linked glycosylation, O-linked glycosylation, combined N- and O-multiple glycosylation, and lipid and glycosylphosphatidylinositol (GPI)-anchored linkage defects, most are associated with the N-linked glycosylation pathway (Ferreira et al., 2018). PMM2-CDG (CDG Ia) is the most common type as it has the highest incidence among CDG patients with a morbidity of 1:100,000 (Altassan et al., 2019a).

Pathogenic mutation of *SSR4* can induce *SSR4*-CDG, whose major clinical symptoms include developmental delay, respiratory distress, feeding difficulty in infancy, appearance deformity (e.g., microcephalus, micrognathia, macrotia, deep-set eyes, etc.), and hypotonia. Some patients experience even more severe symptoms including epilepsy and coagulopathy (Castiglioni et al., 2021). As CDG can affect the functions of multiple organs, diagnosis is challenging. Jaeken et al. (Jaeken et al., 1984) proposed that serum transferrin-isoelectric focusing (Tf-IEF) can screen for CDG. According to the nature of glycosylation defects, they are classified as CDG-I or CDG-II types (Cylwik & Lipartowska et al., 2013; Cylwik & Naklicki et al., 2013). With the availability of molecular diagnostic techniques, it is now known that some CDG patients possess a normal Tf-IEF pattern. Thus, genetic testing is currently the primary means of diagnosing CDG.

According to current knowledge, CDG are typically autosomal recessive disorders. However, in this case, we describe an infant patient with CDG caused by variation in X-linked *SSR4* gene. Our study extends the genotypic and phenotypic spectrum of CDG with the aim of increasing clinicians’ awareness of *SSR4*-CDG.

## 2 Methods

### 2.1 Ethics statement

This study was approved by the Ethics Committee of Children’s Hospital of Soochow University. Informed consent was obtained from the patient’s guardians and family members according to the regulations of the participating institutions in accordance with the Declaration of Helsinki. All family members underwent careful clinical examinations by experienced physicians and laboratory staff at our hospital and provided blood samples for genetic analyses.

### 2.2 Case presentation

**FIGURE 1:**
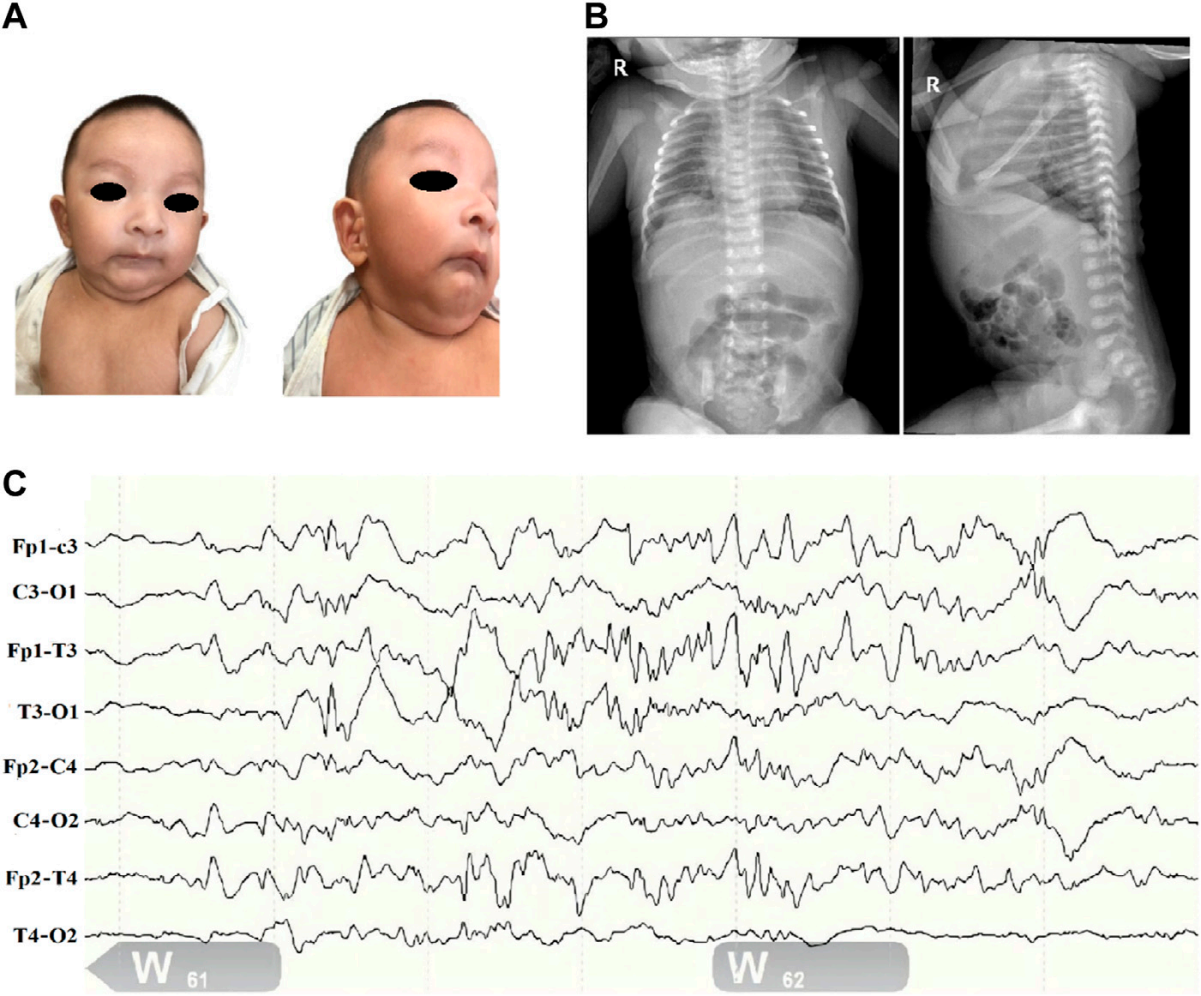
Clinical profile of children with *SSR4*-CDG. **(A)**. Distinctive facial features including microcephaly, deep-set eyes, binaural ear screens with accessible redundancy, small mandibles, and more skin on the neck. **(B)**. Abdominal radiograph report (at 1 day of age): deepened and blurred texture in both lungs; congenital diaphragmatic eventration on the right side. **(C)**. Electroencephalogram (EEG). (1) The background pattern is a normal voltage partial continuum pattern with amplitudes of about 4–39 uv in the QS phase and 7–24 uv in the AS phase, with poor bilateral symmetry. (2) More mature sleep-wake cycle-like activity is seen. (3) No typical epileptiform seizures were seen.

A newborn boy was admitted to our neonatal ward due to respiratory distress occurring 4 h after birth. The infant’s parents were nonconsanguineous; the father had mild cerebral palsy and the mother showed obvious mental deficiency. There was no history of hereditary or contagious disease. The case was delivered spontaneously at 37 weeks of gestation with a birth weight of 2,500 g and a birth length of 45 cm. On admission, the case weighed 2.420 kg, measured 46 cm long with a head circumference of 31 cm, a breathing rate of 62 times per minute, and an unusual face (Figure 1A), including microcephaly, micrognathia, deep-set eyes, and tragus of both ears reaching vegetation. The breath sounds in both lungs were coarse with a few wet rales. The heart rhythm was uniform, the heart sounds were acceptable, a systolic murmur of class II/VI was heard in the precordial region, the abdomen was soft, and the bowel sounds were normal. Furthermore, the case had massive folds on the skin of his neck, asymmetric scapula, and hyperextension of the right knee.

Routine blood and biochemical tests after admission only suggested mild jaundice. Thyroid function, blood and urine genetic metabolic screening and other results were not significantly abnormal. Complete chest and abdominal X ray showed a diaphragmatic eventration on the right side (Figure 1B). Bilateral knee ultrasound showed no significant abnormalities. Ultrasound of the heart showed a septal defect. The 24 h electroencephalogram (EEG) (Figure 1C) showed only frontotemporal spikes and spikes with no typical epileptiform seizures.

After 2 weeks of treatment, including nasal catheter oxygen inhalation (2L/min), phototherapy, and antibiotherapy, the case was discharged with stable respiration and regression of edema in both lower limbs. The chest and abdominal X ray showed diaphragmatic eventration on the right side without retraction or further aggravation at 14 and 21 days of age. Combined with the patient’s clinical phenotype and laboratory tests, congenital hypothyroidism, methylmalonic acidemia, and phenylketonuria were excluded, but rare disorders such as RSS syndrome, which is characterized by feeding difficulties and growth retardation, could not be ruled out. To further clarify the diagnosis, tests such as whole exome assay were carry out.

The case was followed up at our outpatient clinic at 2 months of age, 5 months of age, and 8 months of age. No significant abnormalities were found on the repeated routine blood and biochemical tests. At 2 months of age, the case had persistent feeding difficulties, no recurrent jaundice, no respiratory distress, and no abnormal waveforms on 6 h EEG. At 5 months of age, there were no abnormalities on ultrasound of the bilateral hip and knee joints. Neurobehavioral examinations were completed at 2 months, 5 months, and 8 months of age and revealed general developmental delays in language, social skills, and movement. At 5 months of age, the case started on high-calorie (86 kcal/100 mL) formula. At the last follow-up, when the case was 8 months and 3 days old, he weighed 7.5 kg (weight/age evaluation: lower middle P3-P25), his height was 68.3 cm (height/age evaluation: lower middle P3-P25), his head circumference was 40.5 cm (head circumference/age evaluation: lower < P3), his weight/height evaluation was in the lower middle P3-P25, BMI evaluation was in the lower middle P3-P25, and he showed low muscle tone in both lower extremities (Alberta motor scale score <1%). The case now receives regular rehabilitation training treatment, compliance is good, the family is more satisfied with the therapeutic effect. No adverse events during treatment. Based on genetic counseling, the case’s parents were informed that the patient’s variant was of maternal origin, that newborns of normal pregnancies had a high chance of developing *SSR4*-CDG, and that assisted reproductive technology was feasible if needed.

### 2.3 Whole exome sequencing analysis

Genomic DNA was extracted from peripheral blood and measured using a Nanodrop 2000 (Thermo). Exome sequencing libraries were constructed using the SureSelectXT Human All Exome V6/V6 + UTRs kit (Agilent). Polymerase chain reaction (PCR) products were validated using an Agilent 2,100 Bioanalyzer (Agilent). The primary analysis was performed using built-in software, HiSeq Control Software (HCS), and RTA 2.3 plus. Demultiplexing was performed using bcl2fastq 2.17. Lastly, the raw data from exome sequencing were analyzed by bioinformatics programs. The reads were mapped to the human reference genome (UCSC hg19). The pathogenicity of variants was analyzed according to the American College of Medical Genetics and Genomics (ACMG) standards and guidelines. The average depth of coverage of the target exome region was 117.34-fold and at least 98.23% of the exome was covered.

### 2.4 RNA extraction and quantitative RT-PCR assays

Total RNA was extracted from peripheral blood using TRIZOL (Life science) and the DNA-free kit (Ambion). Three micrograms of RNA per sample were used as the input material for RNA sample preparation. Sequencing libraries were generated using the NEBNext UltraTM RNA Library Prep kit for Illumina (NEB, United States) following the manufacturer’s protocol. The primers for QPCR were designed using Primer five software:

*SSR4*_RT-PCR-F: CAGCCGTTCGGCAGAGAA

*SSR4*_RT-PCR-R: CTT​CGC​ACT​GAA​GGC​CAA​GT

The integrity and quality of cDNA libraries were analyzed using an Agilent 2,100 Bioanalyzer and ABI StepOne plus Real-Time PCR System. The mapping of 100 bp paired-end reads to genes was undertaken using HTSeq v0.6.0 software. All experiments of the three samples were repeated three times, and the Ct values corresponding to each reaction conditions were averaged. GAPDH gene was using as internal reference. A healthy child was selected as a normal control. The relative expression level was calculated by the comparative CT method (ΔΔCT).

### 2.5 Differential gene expression and functional pathway enrichment analysis

cDNA libraries were sequenced using a Illumina HiSeq X ten/NovaSeq system. RNA-seq data were aligned to the UCSC human reference genome using Hisat2 2.0.1 (Kim et al., 2015). Expression of genes (raw count) was quantified by StingTie 1.3.5 (Pertea et al., 2015). Differential gene detection using the Gfold algorithm (Feng et al., 2012). KEGG pathway enrichment analysis of differentially expressed genes was carried out using the R package clusterProfiler (Yu et al., 2012). Only those pathways with adjusted *p*-value <0.05 were considered statistically significant.

## 3 Results

### 3.1 Identification of a novel *SSR4* mutation

**FIGURE 2:**
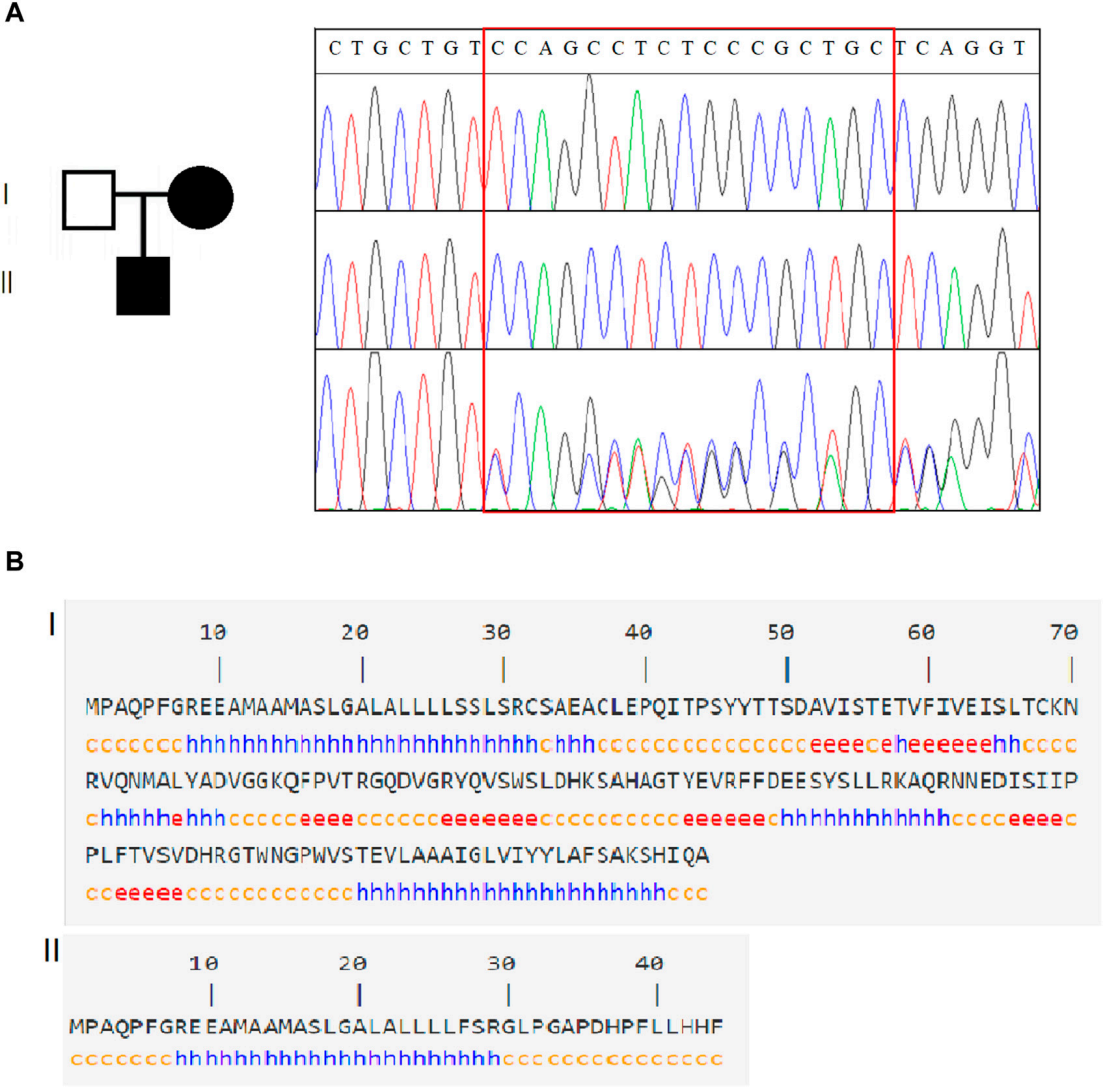
Genetic analysis of *SSR4*. **(A)**. Family information and Sanger sequencing results revealed that *SSR4*-CDG is X-linked recessive. **(B)**. Schematic diagram of the *SSR4* secondary structure. Of the original protein structural domain (140 amino acid structure), only 44 coding amino acid structures were retained after the mutation occurred.

A hemizygous variant c.80_96del in *SSR4* (NM_001204526.1): c.80_96del CCAGCCTCTCCCGCTGC (p.Ser27Phefs*19) was identified in the case (Figure 2A), while his normal mother was a heterozygous carrier, confirming X-chromosome linked recessive inheritance. Notably, it is not included in the HGMD database or the gnomAD database (PM2_Supporting). The variant occurred in exon 2 of the *SSR4* gene transcript. It may cause protein truncation or activate nonsense-mediated mRNA degradation, thereby affecting the function of the protein product encoded by the gene. According to ACMG guidelines, this variant is a suspected pathogenic variant (PVS1+PM2_Supporting). The variant c.80_96del led to an amino acid change of serine with phenylalanine at residue 27 and translation termination after a shift mutation of 18 amino acids (Figure 2B).

### 3.2 *SSR4* expression and aberrant splicing analysis

**FIGURE 3:**
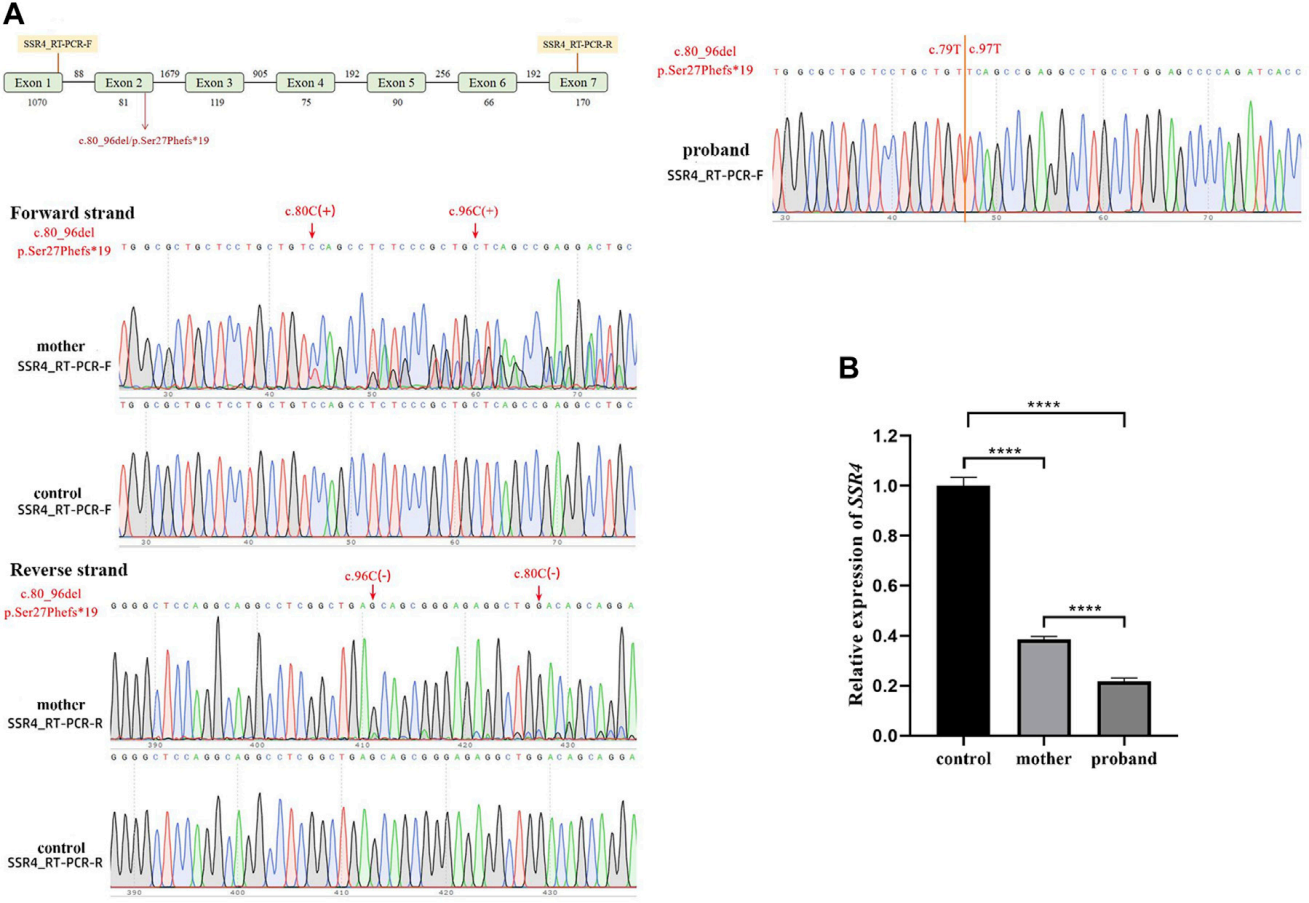
Quantitative RT-PCR assays. **(A)**. Schematic design of *SSR4* cDNA primers and sanger sequencing. **(B)**. mRNA expression. Downregulation of *SSR4* gene expression in a male child with CDG.

The Sanger sequencing results (Figure 3A) the mutant skipped exon 2, indicating that variation (hemizygote) is the possible causal site for gene dysfunction. With the expression level of healthy child as control, *SSR4*-F/R primers test results showed that the expression level of mother was 0.385, and the expression level of patient was 0.217 (Figure 3B).

### 3.3 Differential gene expression and functional pathway enrichment analysis

**FIGURE 4:**
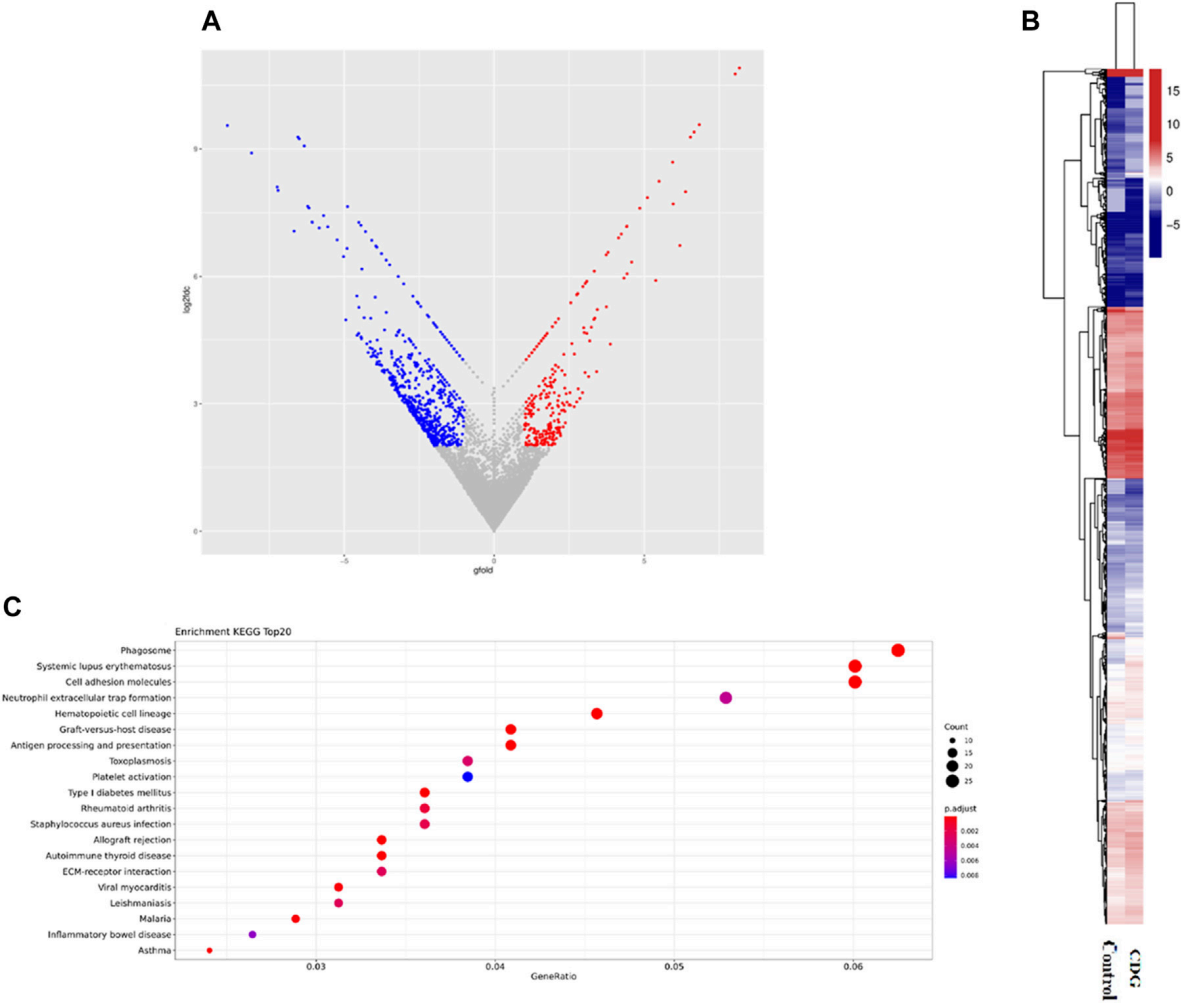
Differential gene expression and functional pathway enrichment analysis. **(A)**. Volcano plot of differentially expressed genes: 536 genes were upregulated in expression (red dots) while 1,078 genes were downregulated in expression (blue dots). **(B)**. Hierarchical clustering heat map of gene expression. **(C)**. Gene set enrichment analysis (GSEA) of the case and controls.

RNA-seq was performed to identify gene expression in the case and healthy control children, and enrichment of releated signaling pathways. According to the results, the patients were present with 1,078 genes downregulated and 536 genes upregulated, among them, *SSR4* gene was significantly downregulated in the case (Gfold: 2.012). (Figures 4A,B). Gene set enrichment analysis (GSEA) of the case and controls revealed significant changes in many signaling pathways of both groups, mostly clustered in hemostasis and coagulation, catabolism, erythrocyte development and homeostasis regulation, and muscle contraction and regulation (Figure 4C).

## 4 Discussion

Glycosylation is an important biological process involving the modification of various proteins and lipids in cells; notably, about 2% of human genes encode glycosylation-related proteins (Apweiler et al., 1999). As CDG can cause multi-organ disorders, genetic analysis was conducted on an infant patient with feeding difficulty, developmental delay, and an unusual face. We identified a new hemizygote variation in his *SSR4* gene: c.80_96del (p.Ser27Phefs*19). Generally, CDGs are due to autosomal recessive inheritance (Freeze et al., 2014b). However, in this case, CDG was due to X-chromosome-linked inheritance from the mother of the patient with a pathogenic gene at chrX:153060189-153060205.

The translocon-associated protein (TRAP) complex, SEC61, and OST complex and glycosylation together participate in the protein translocation process, with the TRAP complex playing a key role. *SSR4*, a subunit of the TRAP complex, is involved in protein translocation across the endoplasmic reticulum membrane and can increase the efficiency of N-linked glycosylation (Hartmann et al., 1993). When the loss of *SSR4* protein impairs the TRAP complex, the resulting defective glycosylation will ultimately lead to CDG (Losfeld et al., 2014; Nagasawa et al., 2007). Using expression tests and aberrant splicing analysis, it was found that the infant’s *SSR4* gene expression was downregulated, and Sanger sequencing (Figures 3, 4) revealed abnormal splicing and exon skipping in some parts of exon 2 (skipping missing parts), indicating that the variation (hemizygote) is the possible pathogenic site.

No significant genotype-phenotype correlation has been found for *SSR4*-CDG due to the limited number of clinical cases. Growth retardation is the most common clinical symptom in *SSR4*-CDG patients (Johnsen et al., 2024). Major clinical symptoms of *SSR4*-CDG include developmental delay, respiratory distress, and feeding difficulty in infancy as well as microcephaly, micrognathia, an unusual face, and hypotonia (macrotia, deep-set eyes, etc.). For some patients, there are even combined symptoms including epilepsy and coagulopathy, while some female patients suffer from mild dysgnosia (Castiglioni et al., 2021). We performed whole-transcriptome on a healthy child and our case and found that most of the many signaling pathways that showed significant changes of healthy child and our case were clustered in hemostasis and coagulation, catabolism, regulation of erythrocyte development and homeostasis, and muscle contraction and regulation. The result suggest that *SSR4* gene may be associated with coagulation abnormalities, hypotonia, gastrointestinal insufficiency and endocrine metabolic disorders, which is consistent with existing reports on the *SSR4*-CDG phenotype (Johnsen et al., 2024).

The patient in this case presented with a global developmental delay, feeding difficulty, microcephaly, micrognathia, and an unusual face. Furthermore, the patient suffered from congenital local diaphragmatic eventration and no retraction was found on X-ray chest film reexaminations, which could be caused by congenital diaphragm dysplasia. More notably, the infant had non-pitting dropsy of the lower limbs. This symptom has never been reported among patients with *SSR4*-CDG, but is more common among PMM2-CDG. Patients that present with hydrops fetalis and neonatal edema usually die between 29 weeks after pregnancy to 3 months after birth (Altassan et al., 2019a), but our patient’s dropsy of the lower limbs disappeared without obvious poor prognosis at the age of 2 weeks. At 8 months of age, neuropsychological developmental tests showed a mean age equivalent to 5.3 months, with overall growth retardation and low muscle tone in both lower limbs. The case’s guardian has good compliance, receives regular rehabilitation training and treatment, and is satisfied with the case’s current condition.

In conclusion, this study has contributed to the literature on mutation loci and clinical phenotypes of *SSR4*-CDG, expanded the variant spectrum of *SSR4*, and contributed to genetic counseling. However, this is a single case report and correlation study, and the correlation between genotype and phenotype is more difficult to determine. Because of objective reasons, we were not able to characterize serum transferrin glycoforms profiles and *SSR4* protein expression in the case. More organism-level studies are needed in the future to elucidate the underlying molecular mechanisms of *SSR4*-CDG.

## Data Availability

The datasets for this article are not publicly available due to concerns regarding participant/patient anonymity. Requests to access the datasets should be directed to the corresponding author.
